# Examining Demographic Variables Associated with Fruit and Vegetable Intake and Skin Yellowness

**DOI:** 10.3390/nu18172804

**Published:** 2026-08-27

**Authors:** Anna K. Jansson, María Gómez-Martín, Erin D. Clarke, Marc T. P. Adam, Clare E. Collins

**Affiliations:** 1 School of Health Sciences, College of Health, Medicine and Wellbeing, The University of Newcastle, Callaghan, NSW 2308, Australia; 2 Nutrition and Metabolic Health Research Program, Hunter Medical Research Institute, New Lambton Heights, NSW 2305, Australia; 3 School of Computer and Information Sciences, University of Newcastle, Newcastle, NSW 2308, Australia

**Keywords:** dietary intake, skin carotenoids, reflectance spectroscopy, nutrition, cross-sectional

## Abstract

**Background/Objectives:** Skin yellowness measured using reflectance spectroscopy can be used as a proxy for ‘skin carotenoid’ concentration and as a valid estimate of fruit and vegetable intake. However, the association between different ethnic backgrounds, age, sex and self-reported skin colour on skin yellowness is yet to be explored. **Methods:** This exploratory cross-sectional study used a convenience sample to assess the relationship between skin yellowness (b*), measured using skin reflectance spectroscopy, and fruit and vegetable intakes across different age groups, sex, self-reported Caucasian and Asian ethnicities and self-reported skin colour. Recruited adults were aged ≥18 years who self-reported as Caucasian or of Asian ethnicity (*N* = 137). Data were collected on skin L*, a*, b* colours using skin reflectance spectroscopy, height, weight, body composition, waist circumference, demographic variables (e.g., age, sex, self-reported skin colour), and dietary intake measured using the online Australian Eating Survey^®^ (AES^®^). Multiple linear regression models were used to investigate factors associated with the dependent variable, skin yellowness (b*). **Results:** Participants were on average 38.6 (*SD* = 17.7) years of age, self-reported as female (61%), Caucasian (77%) and met the recommended fruit intake of two serves/day (mean = 2.1, *SD* = 1.5) but not the recommended vegetable intake of five to six serves per day (mean = 4.4, *SD* = 3.1). There were significant associations between skin yellowness (b*) and several independent variables, including combined fruit and vegetable intake, total carotenoid intake, body fat percentage and self-reported skin colour. **Conclusions:** This study adds additional support for using skin yellowness as a proxy for fruit and vegetable intake when adjusting for self-reported skin colour, age and sex.

## 1. Introduction

Inadequate fruit and vegetable (FV) intakes contribute to approximately 3.9 million deaths globally [1] and are associated with a higher risk of developing several non-communicable diseases [2,3,4,5,6]. Despite the evidence supporting higher FV intakes, only 4% of Australian adults meet recommended FV intake targets [7]. The benefits of adequate FV consumption may partially be due to the higher intakes of phytonutrients, including carotenoids. Carotenoids are lipid-soluble pigments that confer colours such as red, orange and yellow to FV [8]. Importantly, carotenoids have antioxidant and anti-inflammatory properties that contribute to lower risk of developing coronary heart disease, stroke, cancer, type 2 diabetes and all-cause mortality [9,10]. There are approximately 40 different carotenoids in foods consumed in a typical human diet, with about 20 identified as appearing in blood and epithelial cells, with α-carotene, β-carotene, β-cryptoxanthin, lycopene, lutein, and zeaxanthin being the most abundant [11]. When ingested, some carotenoids function as antioxidant nutrients (e.g., to fight oxidative stress) [12], contribute to eye health [13], while β-carotene is converted to vitamin A [14]. Carotenoids not required immediately are stored in all layers of the human skin and adipose tissue [hereafter referred to as skin carotenoids] [15].

The concentration of skin carotenoids depends on several factors in addition to dietary intake, including age, sex, body mass index (BMI), body fat, carotenoid supplements, smoking status and recent sun exposure [16]. Higher levels of skin carotenoids have been associated with perceived healthiness and attractiveness [17], lower prevalence of metabolic syndrome [18], and a lessened adverse impact secondary to ultraviolet radiation exposure [15]. In addition, higher levels of skin carotenoids have also been associated with greater FV intake [19], offering an objective and time-efficient biomarker to help address biases associated with self-reported dietary assessment methods.

There are several ways to measure intakes or accumulation of carotenoids in humans, including [20] carotenoids estimated from self-reported dietary intake (e.g., Australian Eating Survey [AES^®^] [21]), appearance in urine or blood samples [20] and estimating skin yellowness using non-invasive spectroscopy-based measures (e.g., skin reflectance spectroscopy, pressure mediated reflectance spectroscopy and resonance Raman spectroscopy) [19,22]. Measuring skin yellowness using spectroscopy measures has been shown to be a valid proxy of FV intake in diverse human population samples [19]. Skin reflectance spectroscopy measuring skin yellowness (b*), in addition to L* (lightness-darkness) and a* (redness), via a spectrophotometer has been validated against plasma carotenoids in Australian adults as an objective measure of fruit, vegetable and carotenoid intakes [23]. Specifically, the study found that AES^®^ derived carotenoid intakes (i.e., α-carotene, β-carotene, β-cryptoxanthin, lycopene, lutein, and zeaxanthin) were significantly positively associated with skin yellowness across a range of dietary carotenoids (*β* range 0.25–0.46) and total dietary carotenoids (*β*  =  0.35), except lycopene [23].

No studies to date have explored the association between skin yellowness measured using spectrophotometry and food frequency questionnaire (FFQ) among a mix of self-reported Caucasian and Asian ethnicities, different age groups, sex and self-reported skin colour. Therefore, this study aimed to assess the relationship between skin yellowness (measured using skin reflectance spectroscopy) and FV intakes across different age groups, sex, ethnic background and self-reported skin colour.

## 2. Materials and Methods

The current study utilised a convenience sample of adults recruited to a cross-sectional study, conducted in Newcastle, New South Wales, Australia. Data collection took place between October 2023 and June 2025. Ethics approval has been received from The University of Newcastle Human Research Ethics Committee (H-2023-0010). Reporting was guided by the STROBE-NUT guidelines (Supplemental Table S1).

### 2.1. Participants, Eligibility and Recruitment

Eligible participants were (i) ≥18 years old, (ii) proficient in English, (iii) had not used fake tanning products in the week leading up to the assessment, (iv) not a smoker, (v) did not present with any chronic lung condition, and (vi) were not on medication known to increase photosensitivity. Several different recruitment strategies were used to recruit participants from a range of different ethnicities and age groups. These strategies included using flyers posted on University of Newcastle noticeboards, media releases, and social media.

For the current analysis, only participants who met the eligibility criteria and self-reported being of Asian or Caucasian descent were included. This convenience sample was mainly determined by the difficulties recruiting from other ethnic backgrounds.

### 2.2. Measurements

Prior to attending an in-person assessment, participants were administered an online survey, which included several demographic questions and the Australian Eating Survey^®^ (AES^®^) FFQ. During the in-person assessment, height, weight, body composition, waist circumference, and skin L*, a*, b* colours were measured.

#### 2.2.1. Demographic Information

The online survey included questions on participants’ age, sex, gender, self-perception of skin colour and usual sun exposure, medical history, wellbeing, allergies, food security, income management, meal sharing, pregnancy, medication and alcohol consumption. Participants also indicated which ethnicities they identified with, based on a validated list [24], by choosing up to three of the following: Oceania, North-West European, Southern & Eastern European, North African and Middle Eastern, South-East Asian, Southern and Central Asian, Peoples of the Americas, Sub-Saharan African, Aboriginal and/or Torres Strait Islander or other. Self-reported skin colour was measured using a 6-item scale ranging from “Very fair” to “Black” [25].

#### 2.2.2. Dietary Intake Assessment Methods

Dietary intake was evaluated using the AES^®^ FFQ, which has been validated for self-reported fruit and vegetable intakes and diet quality against skin and plasma carotenoids in adults [26,27]. The AES^®^ is an online-administered 120-item FFQ, taking approximately 20–30 min to complete [21]. The consumption of fruits and vegetables was assessed through 11 and 21 questions for fruit and vegetables, respectively. Serving sizes were determined by adding up the total weight of fruits and vegetables consumed, as measured by the AES^®^, and dividing by the standard serving sizes specified in the Australian Guide to Healthy Eating (i.e., 150 g for fruit and 75 g for vegetables) [21].

From the AES^®^, the Australian Recommended Food Score (ARFS) diet quality scores for fruit and vegetable sub-scales, total score and ‘other’ (i.e., total ARFS score minus fruit and vegetable score) were also used. The ARFS has been validated against skin carotenoids [27] and is calculated based on a subset of 70 questions related to core nutrient-dense foods, with 20 questions related to vegetable intake (max score = 21), 12 questions related to fruit (max score = 12) and the remainder of questions are related to other food groups (e.g., protein foods—meat/flesh, vegetarian sources of protein, breads and cereals, dairy). The total ARFS ranges from zero to 73.

Carotenoid (i.e., α-carotene, β-carotene, lycopene, cryptoxanthin, combined lutein/zeaxanthin and total carotenoids) intake was calculated using the AUSNUT 2011-2013 database [28] and the US Department of Agriculture and the National Cancer Institute (USDA-NCI) [29]. These methods have been described in more detail elsewhere [30].

#### 2.2.3. Skin Yellowness Measure Using Skin Reflectance Spectroscopy

A skin reflectance spectroscopy device (CM700D, Konica Minolta, Tokyo, Japan) was used to measure skin yellowness. Skin reflectance spectroscopy is a validated method to assess dietary carotenoids, fruit and vegetable intakes [26,27]. For each participant, a white calibration of the skin reflectance spectroscopy device was conducted. Then, skin colour (CIE L*a*b* values, where positive L*, a* and b* values represent lightness, redness, and yellowness, respectively) was measured on four different sanitised body regions (i.e., forehead, palm of the left hand, inner left arm, and under left foot) using the same methods for all participants. Body locations were selected according to anatomical landmarks using the ISAK international standards for anthropometric assessment [31]. These sites were chosen as they are less exposed to sunlight. The measurements were repeated three times at each body region, and the data were processed using Spectramagic NX (version 3.40). For each body region, the mean of the three repeated measures was calculated; the total average was then derived from the four regional means. The researchers performing measures had received training in using the device from a senior assessor.

#### 2.2.4. Other In-Person Measures

Height was measured using a portable stadiometer, correct to 0.1 cm, using the stretch stature method. Body composition (i.e., weight, fat mass and lean mass) was measured using the InBody720 Body Composition Analyzer (Biospace Co., Ltd., Seoul, Republic of Korea). Participants were instructed to wear light clothing and remove socks, shoes and metal items. Waist circumference was measured using a flexible tape measure at the narrowest part of the abdomen, between the lower costal border and the top of the iliac crest, perpendicular to the long axis of the trunk. The average of two measurements to the nearest 0.1 cm was used in the analysis.

### 2.3. Statistical Analysis

Data analyses were undertaken using R (Version 4.4.3). Data are presented as percentages or means and standard deviations (SD). To address the presence of outliers, we applied a standard winsorization, where values that were more than 1.5 IQR below Quartile 1, or more than 1.5 IQR above Quartile 3, were considered outliers. Values below the lower threshold were set to the lower threshold value, and values above the upper threshold were set to the upper threshold value. This was applicable to the following variables: total carotenoids, α-carotene, β-carotene, cryptoxanthin, lutein/zeaxanthin, ARFS (other), fruit intake (serves/d), vegetable intake (serves/d), total energy intake, fat intake, and lightness-darkness (L*) (see Supplemental Table S2). This approach maintained the sample size while reducing the influence of extreme observations on subsequent analyses.

Prior to conducting the regression analyses, several transformations were performed to improve interpretability of some variables that could not be interpreted at value zero (i.e., body fat percentage, skin lightness-darkness, total energy and fat intake) or exhibited low very small values (e.g., carotenoids). All individual and total carotenoids, total energy intake, and skin lightness-darkness (L*) were transformed using z-standardisation. Body fat percentage was re-scaled based on the minimum body fat percentage needed for essential physiological functions (i.e., 3% for males and 12% for females) [32]. The minimum essential body fat percentage is believed to be higher in females due to childbearing and hormonal functions [32].

Dummy variables were created for self-reported skin colour (i.e., fair, light olive, dark olive/brown), where ‘very fair’ was the reference value. Dark olive and brown were combined due to the low sample size. Dummy variables were also created for sex, where male was the reference value, and age group, where those aged <35 were the reference group. Age was dichotomised into two groups: <35 years versus ≥35 years, as this created the most even split of the dataset and those under <35 are considered ‘young adults’. For the dummy season variable (dummy winter/autumn), winter and autumn were combined due to the low number of data collected within these seasons and summer/spring was the reference value. The dummy Asian variable was created based on participants choosing an ‘Asian’ ethnicity category, according to the ASCCEG [24]. If a participant ticked an Asian category such as South-East Asia or North-East Asia (even if they had also chosen another category), they were classified in the “self-reported Asian” category.

To investigate factors associated with skin yellowness (b*) (dependent variable), four different multiple linear regression models were conducted. The first model (1) only considered demographic (i.e., self-reported skin colour, sex, age group [<35 versus ≥35], body fat percentage, skin lightness-darkness (L*), dietary fat intake (grams)) and seasonal (dummy winter/autumn) variables, known to influence skin yellowness. The second model (2), additionally took into account fruit and vegetable intake (i.e., serves of fruit and vegetable per day), while the third model (3) alternatively considered the ARFS scores for fruit, vegetable and ‘other’ (i.e., the total ARFS score minus the ARFS subscores for fruit and vegetable). Finally, the fourth model (4) considered total dietary carotenoids. For each model set, we evaluated model fit using adjusted R-squared values. The incremental contribution of each block of variables (models 2–4) was assessed by comparing nested models within each set. In the supplemental material, the independent fruit and vegetable intake (model 2), total ARFS score (model 3), ARFS fruit and ARFS vegetable (model 4) and all independent dietary carotenoids (i.e., α-carotene (μg), β- carotene (μg), β-cryptoaxanthin (μg), lycopene (μg), lutein and zeaxanthin (μg); model 5) were considered instead. A number of sensitivity analyses were also performed, including re-evaluating the main models using the non-winsorized data (Supplemental Table S4) and exploring the potential influence of collinearity between total energy intake and fat intake (Supplemental Tables S5 and S6). Significance was set at *p*-value < 0.05.

## 3. Results

**Figure 1:**
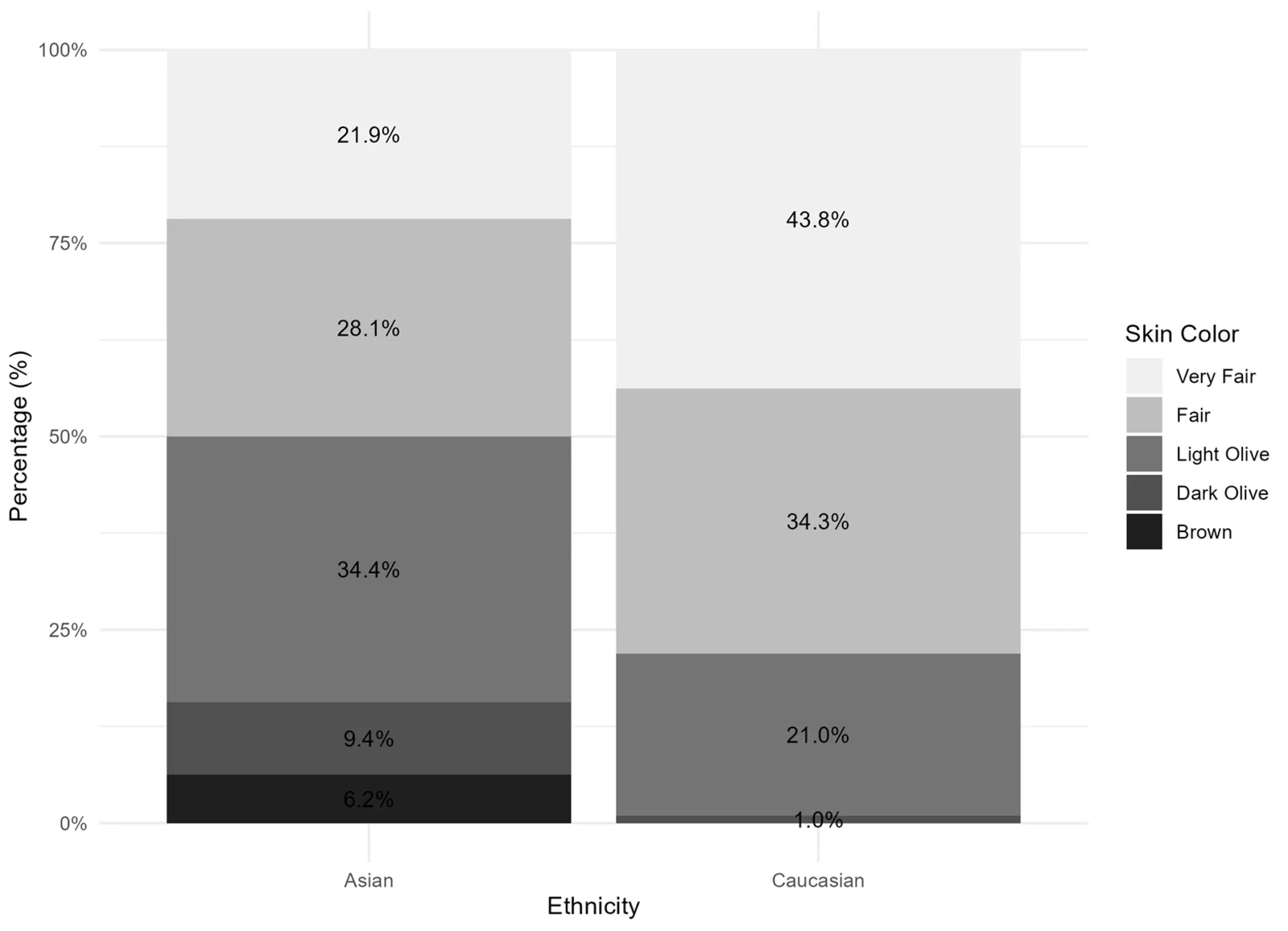
Proportion of self-reported skin colour by self-reported ethnicity (*N* = 137).

Of the 167 participants recruited, 30 did not complete the in-person assessment and were excluded, yielding a final sample of 137 participants. Included participants were, on average, 38.6 (*SD* = 17.7) years old, and the majority identified as female sex (60.6%) and Caucasian (76.6%). Participants, on average, met the recommended daily intake of two serves of fruit (mean = 2.1, *SD* = 1.5) but not the recommended five to six serves of vegetables per day (mean = 4.4, *SD* = 3.1). The majority of Caucasian participants (43.8%) reported having a very fair skin tone, while most of Asian participants reported having a light olive skin tone (34.4%) (Figure 1). More demographic characteristics are outlined in Table 1.

Table 2 reports the main results, with additional analyses presented in Supplementary Tables S3 and S4. For self-reported skin colour, ‘light olive’ had a significantly higher b* value across all four models (coefficients between 0.83–0.93) compared to ‘very fair’, while ‘dark olive/brown’ had a significantly higher b* value in model 1 (1.39, *SE* = 0.66), model 2 (1.34, *SE* = 0.64) and model 4 (1.42, *SE* = 0.64), but not when ARFS scores (model 3) were adjusted for. There was a significant association between skin yellowness (b*) and body fat percentage (coefficients between −0.07 and −0.08) and skin lightness-darkness (coefficients between −0.65 and −0.72) across all models. There were no significant associations between skin yellowness (b*) and ‘fair’ and ‘very fair’ skin colour, age groups (i.e., <35 versus ≥35 years), sex (i.e., males vs. females) or season (i.e., winter/autumn vs. summer/spring) in any models (*p*-value > 0.05). There were also no significant associations between total energy intake or fat intake and skin yellowness (b*) across all models (*p*-value > 0.05). Only a limited proportion of variables were winsorized in the main models (Table 2), including 6% of total carotenoid, 4% of fat, and 2% of fruit and vegetable serves data points. A sensitivity analysis of the main models, including the original non-winsorized data, can be found in Supplemental Table S4. Sensitivity analyses were also performed by re-examining all main models by excluding fat and separately excluding total energy intake, to examine the influence of multicollinearity between the two variables. It should also be noted that the main models only account for 38–41% of the variance in the data, with only modest increases in adjusted R^2^ when accounting for dietary variables.

For the dietary intake, there were significant associations between fruit and FV intake and skin yellowness (b*), where each fruit and FV served increased skin yellowness (b*) by 0.30 (*SE* = 0.12) and 0.10 (*SE* = 0.04), respectively (see Supplemental Table S7). There was also a significant association between total carotenoid intake and skin yellowness (b*), where each SD increase in total carotenoids was associated with an increase in skin yellowness (b*) of 0.41 (*SE* = 0.15). For individual carotenoids, beta carotene (1.18, *SE* = 0.55) was the only one that was significantly associated with skin yellowness (b*) (see Supplemental Table S3). There was no significant relationship between ARFS other or ARFS FV and skin yellowness (b*) (*p*-value > 0.05). However, when ARFS total and ARFS fruit were considered, for each 1-point increase, there was a significant 0.03 (*SE* = 0.01) and 0.13 (*SE* = 0.05) unit increase in skin yellowness (b*), respectively (see Supplemental Table S7).

In Supplemental Table S8, alternative regression models are presented, which consider self-reported Asian or Caucasian ethnicity, instead of skin colour. These models found that self-reported Asian ethnicity was associated with a higher skin yellowness (b*) (coefficients between 1.09 and 1.50) compared to Caucasian ethnicity. Body fat percentage and skin lightness (L*) also remained significantly associated with skin yellowness (b*) across all four models, −0.06 to −0.07 and −0.65 to −0.72, respectively. There were also significant associations between FV intake (0.15, *SE* = 0.04), ARFS other (0.07, *SE* = 0.03), ARFS FV (0.05, *SE* = 0.02) and total carotenoids (0.53, *SE* = 0.15) with skin yellowness (b*), when ethnicity was considered.

## 4. Discussion

The current exploratory study identified significant associations between skin yellowness (b*) measured using skin reflectance spectroscopy and several independent variables including combined fruit and vegetable intake, total carotenoid intake, body fat percentage, self-reported skin colour, and self-identified Asian ethnicity (Supplemental Table S8). These findings suggest that both intrinsic (e.g., ethnicity and skin colour) and extrinsic (e.g., dietary intake) factors contribute to skin yellowness and may therefore be associated with skin carotenoid deposition.

In the current study, fruit intake met recommended daily targets (≥2 servings/day) [33], which may partially explain the significant associations observed with fruit intake, as well as combined fruit and vegetable (FV) intakes and skin yellowness. In contrast, vegetable intake alone did not show a significant association with skin yellowness, with participants in the current sample having mean intake below recommended targets (4.4 vs. 5 to 6 served/day, respectively) [33]. These findings align with previous studies that have reported similar patterns, where FV intake was significantly associated with skin yellowness, but vegetable intake alone was not [26,34]. Interestingly, a more recent study that reported slightly higher vegetable daily intakes of approximately one serve greater (369 g/day vs. 322.5 g/day in the current study) reported stronger Kappa coefficients for vegetable intake and skin yellowness than for either fruit or combined FV intake [23]. Further research is needed to establish the dose of vegetable intake required to produce detectable increases in skin yellowness.

The lack of a significant association in the current study between skin yellowness and vegetable intake could also be attributed to variability in carotenoid bioavailability, which is influenced by factors including FV types, preparation methods (e.g., raw vs. cooked), and co-consumption with dietary fat (e.g., oils). Although the AES^®^ FFQ has previously demonstrated effectiveness in assessing dietary carotenoid intake, we previously reported that foods rich in carotenoids like mango, papaya, lentils, spinach, broccoli and corn accounted for the greatest variation in skin yellowness [34], without assessing how these foods are prepared or consumed, which can significantly affect carotenoid absorption. Additionally, some vegetables included in the AES^®^ (e.g., potatoes, cauliflower) contain low levels of carotenoids and are unlikely to contribute meaningfully to skin yellowness, while others contain large amounts of specific carotenoids such as β-carotene (e.g., mango, papaya, lentils, spinach, broccoli), lutein-zeaxanthin (e.g., lentils, spinach, broccoli, corn), and α-carotene (e.g., corn) [35,36]. These carotenoids accumulate in both the dermis and epidermis [37] and are associated with skin yellowness [38,39,40]. Future studies should evaluate the association of consuming vegetables with high versus low carotenoid content, and also consider how they are prepared and consumed. Promoting increased vegetable intake, vegetable variety, and meeting dietary recommendations remains essential for optimising health outcomes [41,42] as well as carotenoid intakes [27,43].

Carotenoids have antioxidant and anti-inflammatory properties that contribute to a reduction in oxidative stress and prevention of lipid peroxidation, while acting as co-factors in cellular signalling pathways related to apoptosis, all of which are relevant to high body fat percentage, which is pro-inflammatory [44,45]. As anticipated, current findings were aligned with previous studies that have identified an inverse relationship between carotenoid concentrations and higher body fat percentage [46]. These associations may be due to an association between excess adipose tissue and oxidative stress, which reduces circulating carotenoids and hence decreases deposition in the skin [47]. However, dissimilar to these studies, the current results found no association between skin yellowness and sex. This may be explained by the re-scaling of body fat percentage to account for sex differences, as the minimum body fat percentage in females is higher to account for physiological functions versus males [32]. Future studies are therefore recommended to account for sex-based differences in minimal body fat percentage when evaluating associations with skin yellowness and/or carotenoids.

Self-reported skin tone was consistently associated with skin yellowness across all models, with darker skin tones corresponding to higher levels of skin yellowness when adjusted for FV intake. This may be partially explained by the significant association between skin lightness-darkness (L* values) and yellowness (b* values) measured using skin reflectance spectroscopy as identified in the current study, as well as in previous studies [27,48], rather than ethnicity itself. As shown in Supplementary Table S8, Asian ethnicity was significantly associated with higher skin yellowness, with increases of 1.09, 1.27, 1.50, and 1.28 units across the four models, respectively. However, it is possible that this effect is better explained by skin colour, which varied between the two ethnicities. For example, 22% and 34% of Asian participants reported having very fair and light olive skin, compared to only 44% and 21% of Caucasians, respectively. Future studies should continue to evaluate whether it is ethnicity itself or skin colour that is predominantly driving the effects on skin yellowness.

Skin carotenoid concentrations generally decrease with age, partly due to increased oxidative stress and age-related lifestyle factors, such as illness and sun exposure, where lower skin carotenoid levels are linked to deeper wrinkles and more pronounced signs of skin aging [49,50,51]. Skin carotenoids have also been proposed as an alternative biomarker for cardiovascular health [52]. While our study did not find an association between skin yellowness and age groups (<35 vs. ≥35 years), it is possible that addressing this in greater depth requires a larger sample of older age groups. Particularly as age was dichotomised and categorised as >35 years, this may miss relationships unique to older adults who are classified as ≥65 years. However, the current sample included only a small number of people aged 65+ years (*n* = 20), which is not sufficient to investigate systematic differences in skin yellowness for older adults. Therefore, future studies should evaluate the association of more specific age groups on skin yellowness.

### Strengths and Limitations

Key strengths of the current study are the inclusion of an age and ethnically diverse sample, and the re-scaling of body fat percentage to account for sex differences. Limitations include a relatively small sample size, using a convenience sample, self-report of skin colour, and a low number of participants who self-report their skin colour as dark olive or brown. Given the small sample size, we were unable to include both ethnicity and self-reported skin colour in the same model due to multicollinearity. Skin colour was self-reported using a 6-item scale rather than measured objectively. Consequently, some misclassification may have occurred due to differences in participant perception and interpretation of skin colour categories. Other limitations include using self-reported dietary assessments to estimate dietary intake and the results having limited generalizability.

## 5. Conclusions

The current study found that skin yellowness (b*) was significantly associated with combined fruit and vegetable intake and fruit intake alone in models adjusted for self-reported skin colour, age group and sex. Of key interest was the significant association between skin yellowness (b*) and body fat percentage, but not for sex. Future studies should account for sex differences when evaluating body fat percentage and assess the relationship with skin yellowness in larger population samples across different age groups, skin tone and ethnic diversity.

## Figures and Tables

**Table 1:** Baseline characteristics of included participants.

*N* = 137	Mean/*n*	*SD*/%
**Socio-demographic characteristics**		
Age (years)	38.6	17.7
***Age group (n, %)***		
Aged ≥35 years	64	46.7%
Aged <35 years	73	53.3%
***Sex (n, %)***		
Female	83	60.6%
Male	54	39.4%
***Gender (n, %)***		
Woman	82	59.9%
Man	52	38%
Non-binary/gender diverse	3	2.2%
Self-reported Asian ethnicity	32	23.4%
BMI (kg/m^2^)	24.4	4.6
Body fat percentage (%)	26.2	9.9
Waist circumference (cm)	81.9	12.6
**Season of assessment (*n*, %)**		
Autumn	18	13.1%
Spring	74	54%
Summer	40	29.2%
Winter	5	3.6%
**Self-reported ethnicity (*n*, %)**		
Oceanian	61	44.5%
Northern/Western European	45	32.8%
Southern/Eastern European	9	6.6%
South-East Asian	15	10.9%
North-East Asian	14	10.2%
South-Central Asian	5	3.6%
Americas	3	2.2%
Australian Aboriginal/Torres Strait Islander	6	4.4%
Reported both Caucasian and Asian ethnicity	3	2.2%
**AES Dietary Intake (mean, *SD*)**		
ARFS (maximum 73)	33.1	11.8
ARFS vegetables (maximum 21)	11.7	5.1
ARFS fruit (maximum 12)	5.4	3
Fruit (g/day)	293.8	202.6
Vegetables (g/day)	329	234.3
Total carotenoids (μg/day)	17,246.6	10,876.7
α-carotene (μg/day)	2140	2009.2
β-carotene (μg/day)	7473.8	5803.7
β-cryptoxanthin (μg/day)	472.3	433.9
Lutein/zeaxanthin (μg/day)	2608.7	2149.8
Lycopene (μg/day)	4551.8	2436
**Multivitamin use (*n*, %)**		
Currently taking multivitamins	55	40.1%
2 or less	16	29.1%
3 to 5	13	23.6%
6 to 10	14	25.5%
10 or more	12	21.8%
**Skin colouration reflectance spectroscopy (mean, *SD*)**		
Total Lightness (L*)	63.9	3
Total Redness (a*)	9.4	1.4
Total Yellowness (b*)	16.9	1.8
**Self-reported skin colour (*n*, %)**		
Very fair	53	38.7%
Fair	45	32.8%
Light olive	33	24.1%
Dark olive	4	2.9%
Brown	2	1.5%

For continuous variables: Mean and SD. For categorical variables: *n* and %.

**Table 2:** Linear regression evaluating the association between self-reported skin colour and fruit and vegetable intakes, ARFS fruit and vegetables, ARFS other, on total skin yellowness.

	Dependent Variable: Skin Yellowness b*							
	(1)		(2)		(3)		(4)	
	Regression Co-Efficient	SE	Regression Co-Efficient	SE	Regression Co-Efficient	SE	Regression Co-Efficient	SE
Dummy: Fair	0.42	(0.29)	0.41	(0.29)	0.39	(0.29)	0.42	(0.28)
Dummy: Light olive	0.93 **	**(0.34)**	0.83 *	**(0.34)**	0.86 *	**(0.34)**	0.84 *	**(0.34)**
Dummy: Dark olive/brown	1.39 *	**(0.66)**	1.34 *	**(0.64)**	1.23	(0.65)	1.42 *	**(0.64)**
Dummy: Female sex	0.21	(0.27)	0.15	(0.27)	−0.01	(0.29)	0.14	(0.27)
Dummy: Age ≥ 35	−0.12	(0.27)	−0.32	(0.28)	−0.31	(0.28)	−0.23	(0.27)
Body fat percentage (scaled)	−0.08 ***	**(0.01)**	−0.07 ***	**(0.01)**	−0.07 ***	**(0.01)**	−0.07 ***	**(0.01)**
Skin lightness L* (z-score)	−0.65 ***	**(0.15)**	−0.70 ***	**(0.15)**	−0.67 ***	**(0.15)**	−0.72 ***	**(0.15)**
Total energy intake (z-score)	0.39	(0.38)	0.10	(0.39)	0.01	(0.41)	0.08	(0.39)
Fat (gram/d)	−0.01	(0.01)	−0.00	(0.01)	−0.00	(0.01)	−0.00	(0.01)
Dummy: season winter/autumn	0.24	(0.34)	0.20	(0.33)	0.28	(0.33)	0.19	(0.33)
Fruit and vegetable intake (serves/d)			0.10 *	**(0.04)**				
ARFS other (max score 40)					0.05	(0.03)		
ARFS fruit and vegetables (max score 33)					0.02	(0.02)		
Total carotenoids (z-score)							0.41 **	**(0.15)**
Constant	18.25 ***	**(1.00)**	17.57 ***	**(1.02)**	16.92 ***	**(1.15)**	18.18 ***	**(0.98)**
Observations	137		137		137		137	
R^2^	0.42		0.45		0.45		0.46	
Adjusted R^2^	0.38		0.40		0.40		0.41	
Residual Std. Error	1.40 (*df* = 126)		1.37 (*df* = 125)		1.38 (*df* = 124)		1.36 (*df* = 125)	
F Statistic	9.30 *** (*df* = 10; 126)		9.32 *** (*df* = 11; 125)		8.42 *** (*df* = 12; 124)		9.53 *** (*df* = 11; 125)	

ARFS, Australian Recommended Food Score. ARFS other, the total score minus the score for fruit and vegetables. D, day. SE, standard error. * *p*-value < 0.05; ** *p*-value < 0.01; *** *p*-value < 0.001. Models show regression coefficients with skin yellowness b* as the dependent variable. All models include the same baseline covariates (dummies for: self-reported skin colour, sex, age group and season, and body fat percentage, skin lightness L*, total energy intake and fat intake). Model 2 adds fruit and vegetable intake; Model 3 adds ARFS other and ARFS fruit & vegetables score; Model 4 adds total carotenoids. Z-scores were calculated using the sample mean and standard deviation. Some variables were scaled using z-scores (skin lightness L*, total carotenoids, and total energy intake) and the minimum body fat percentage cut point needed for essential bodily functions (3% for males and 12% for females).

## Data Availability

The raw data supporting the conclusions of this article will be made available by the authors on request.

## Supplementary Materials

The following supporting information can be downloaded at: https://www.mdpi.com/article/10.3390/nu18172804/s1, Figure S1: Correlation matrix showing relationship between serves of fruit and/or vegetables, and dietary intake excluding fruit and vegetable intake (i.e., arfs_other); Table S1: STROBE-nut: An extension of the STROBE statement for nutritional epidemiology; Table S2: Number of variables that were winsorized; Table S3: Sensitivity analysis of the main regression models with influential observations excluded; Table S4: Linear regression evaluating the association between self-reported skin colour and fruit and vegetable intakes, ARFS fruit and vegetables, ARFS other on total skin yellowness (non-winsorized data); Table S5: Linear regression evaluating the association between self-reported skin colour and fruit and vegetable intakes, ARFS fruit and vegetables, ARFS other on total skin yellowness. Sensitivity analysis including total energy intake, with fat intake excluded; Table S6: Linear regression evaluating the association between self-reported skin colour and fruit and vegetable intakes, ARFS fruit and vegetables, ARFS other on total skin yellowness. Sensitivity analysis including fat intake, with total energy intake excluded; Table S7: Linear regression including fruit and vegetable intake, scores for ARFS total, ARFS fruit, ARFS vegetables and individual carotenoids; Table S8: Linear regression with self-reported ethnicity, without skin colour. Reference [53] is cited in the supplementary materials.

## List of Abbreviations

ARFS—Australian Recommended Food Score; AES^®^—Australian Eating Survey^®^; BMI—body mass index; FFQ—food frequency questionnaire; FV—fruit and vegetables.
